# Antithrombotic therapy in lower extremity peripheral artery disease patients with venous thromboembolism: a nationwide cohort study

**DOI:** 10.1016/j.rpth.2025.103291

**Published:** 2025-12-09

**Authors:** Jamilla Goedegebuur, Elena Butera, Qingui Chen, Behnood Bikdeli, Walter Ageno, Roberto Pola, Angelo Porfidia, Stefano Barco, Thijs E. van Mens, Joost R. van der Vorst, Suzanne C. Cannegieter, Frederikus A. Klok

**Affiliations:** 1 Department of Thrombosis and Haemostasis, Leiden University Medical Center, Leiden, The Netherlands; 2 Department of Clinical Epidemiology, Leiden University Medical Center, Leiden, The Netherlands; 3 Thrombosis Clinic, Department of Ageing, Orthopedic, and Rheumatologic Sciences, Fondazione Policlinico Universitario A. Gemelli IRCCS, Università Cattolica del Sacro Cuore, Rome, Italy; 4 Division of Cardiovascular Medicine, Brigham and Women’s Hospital/ Harvard medical School, Boston, Massachusetts, United States of America; 5 Yale-New Haven Hospital Center for Outcomes Research and Evaluation (CORE), Connecticut, Massachusetts, United States of America; 6 Department of Medicine, University of Padua, Padua, Italy; 7 Department of Angiology, University Hospital Zurich, Zurich, Switzerland; 8 Center for Thrombosis and Hemostasis, University Medical Center Mainz, Mainz, Germany; 9 Department of Vascular Surgery, Leiden University Medical Center, Leiden, The Netherlands

**Keywords:** anticoagulants, peripheral arterial disease, platelet aggregation inhibitors, treatment outcome, venous thromboembolism

## Abstract

**Background:**

Antithrombotic therapy (ATT) is recommended for patients with symptomatic lower extremity peripheral arterial disease (PAD). Optimal management of “breakthrough” venous thromboembolic events (VTEs) in these patients remains unclear. This study aims to describe current ATT prescription patterns in PAD patients before, during, and after VTE (treatment) and subsequent clinical outcomes.

**Methods:**

Using Dutch nationwide data, this cohort study identified patients with a reimbursement code for PAD between 2013 and 2021. Within this source population, patients with an International Classification of Diseases-10 code for VTE were identified and followed from VTE date until end-of-study date or death, whichever occurred first. ATT prescriptions, determined from pharmacy records, were mapped in 3 timeframes: 3 months before VTE, during VTE treatment, and 4 to 12 months after VTE.

**Results:**

Patients with PAD (*N* = 1866) and a concurrent VTE were included, with a mean age of 71.7 years and a median survival of 3.8 years. Before the VTE, 64% used antiplatelet therapy only, in contrast to 12% after VTE treatment. During the VTE treatment period, direct oral anticoagulants were the most frequently dispensed ATT type (40%), whereas 27% received anticoagulation plus antiplatelet therapy during this time. The one-year cumulative incidences of arterial thromboembolic events and clinically relevant bleedings were 6.1% (95% CI: 4.9-7.3) and 1.8% (95% CI, 1.2-2.4), respectively.

**Conclusion:**

Immediate treatment of VTE and long-term ATT use in PAD patients were heterogeneous in our cohort. A substantial proportion of patients had prescriptions for both antiplatelet therapy and anticoagulation during the VTE treatment period. Our findings highlight the need for consensus on this complex clinical dilemma.

## Essentials

• It is unclear how to treat patients with PAD and VTE.
• Treatment patterns and outcomes in patients with concurrent PAD and VTE were assessed.
• VTE treatment patterns in PAD patients were very heterogeneous.
• Many patients with PAD received both antiplatelet and anticoagulation therapy after a VTE.

## Graphical abstract

**Figure:**
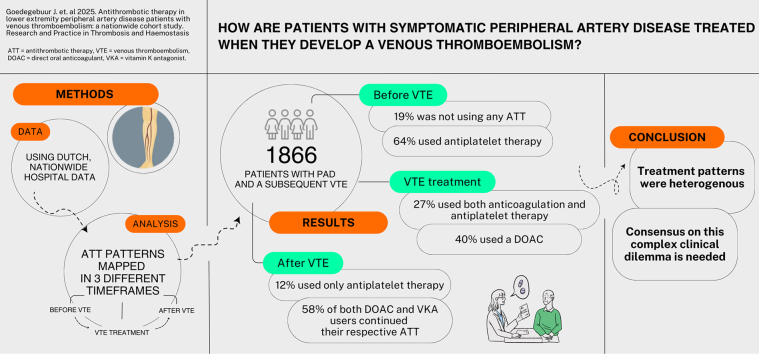

## 1 Introduction

Peripheral arterial disease (PAD) in the lower extremities has a high prevalence in the global population, rising to 20% in persons older than 70 years [1]. For over 20 years, the association between PAD and venous thromboembolism (VTE) has been recognized [[2], [3], [4], [5]]. Studies have not yet yielded conclusive results [6,7], but explanations for the association are thought to be endothelial damage [2], shared risk factors such as age, higher body mass index, diabetes mellitus, and impaired mobility due to claudication or hospitalizations [[8], [9], [10]].

While there are some parallels with the treatment of patients with atrial fibrillation and coronary artery disease [11,12], little is known about the risk and optimal VTE treatment in the general PAD population. Most studies investigating VTE risk or treatment in patients with PAD focus on VTEs occurring after PAD-related procedures [[13], [14], [15]]. This lack of evidence results in a complete lack of guidance on the treatment of VTE in patients with PAD, especially on the optimal antithrombotic therapy (ATT, ie, anticoagulants and antiplatelet agents) after the VTE treatment period has passed, probably leading to heterogeneity in practice patterns [[16], [17], [18]]. To investigate practice patterns and patients’ outcomes, observational cohort study designs are necessary, as these type of research questions cannot be easily evaluated through randomized controlled trials, since in such designs inevitably only selected patient groups will be included, and reaching a sufficient sample size in a reasonable time period would be challenging. The goal of this study was therefore to describe (1) current prescription patterns in patients with PAD who develop VTE, (2) how ATT is further modified after the VTE treatment period (lasting generally 3 to 6 months) has passed, and (3) incidences of arterial thromboembolic events, clinically relevant bleedings, and major adverse limb events (MALE) after VTE occurred.

## 2 Methods

### 2.1 Data sources

In our study, Dutch, nationwide electronic healthcare record data were used from Statistics Netherlands (“Centraal Bureau voor de Statistiek” (CBS), a Dutch governmental institution that gathers and links de-identified individual data from various nationwide data sources) from January 1, 2013 to January 1, 2022 (Supplemental Material section 1). This database covers all Dutch residents’ admissions to general and academic hospitals and two categorical hospitals in The Netherlands (details about the used data have been previously published [19]). Linking between datasets occurs using a person’s individual, pseudonymized number. Extracted data include demographics, data on registered diagnoses during Dutch hospital admissions (using the 9th and 10th revised version of the International Classification of Disease, [ICD-10]), outpatient medication prescriptions, and deaths. Data on medication prescriptions concern Anatomical Therapeutical Chemical classification (ATC5) codes of dispensed outpatient medication prescriptions that were reimbursed under the basic health insurance in The Netherlands. Of note, this includes prescriptions for acetylsalicylic acid as well. These data were complemented with data on diagnosis treatment combinations (DBCs) from Vektis, a company that handles data of all health care reimbursements in The Netherlands. A DBC is a 9-digit code reflecting a package of care for a specific condition, which is used for the reimbursement of the provided care’s costs from the health insurance, including both out- and inpatient care. Patients with lower extremity PAD were identified using the DBCs for PAD stage II, III, and IV, as they are mostly treated in the outpatient clinic and could not be completely captured using data from hospital admission diagnoses only. A detailed list of all variables with the codes used for data extraction is described in Supplemental Material section 2. This study was conducted in accordance with the Declaration of Helsinki. Approval for the study was granted by the Science Committee of the Department of Clinical Epidemiology of the Leiden University Medical Center. Since deidentified registry data were used, according to Dutch regulations, ethical approval under the Medical Research Involving Human Subjects Act (WMO) is not required, and neither is informed consent.

### 2.2 Study population

First, the source population was composed by selecting all adult patients with a DBC code for symptomatic PAD (ie, Fontaine stage II, III, or IV) between January 1, 2013, and January 1, 2022. Next, within this population, patients who were diagnosed and treated for VTE in a hospital were selected (either during hospitalization or on the emergency department, where patients were admitted for at least 4 hours), at any time since the day PAD had been diagnosed. If patients had multiple DBC codes of PAD registered prior to their VTE (possibly indicating different severities of the disease), the most recent DBC code (indicating the most recent disease severity) prior to the VTE was extracted. The date of the VTE was defined as the index date. Comorbidities present on the index date were identified by the presence of a hospital discharge code within three years before the index date, using any primary or secondary diagnosis code (ICD-9 or ICD-10) or medical procedure code (Supplemental Material section 2). Follow-up started from the index date onwards until the end of data collection (January 1, 2022) or death, whichever came first. Given that the study utilized nationwide data, loss to follow-up was expected to be rare, as it could only be related to emigration. A sensitivity analysis was conducted in the cohort, excluding patients with a history of atrial fibrillation, to determine the incidence in a population without this prevalent additional indication for therapeutic anticoagulation.

### 2.3 Study design

Three different treatment periods were defined: (1) the pre-VTE treatment period, (2) VTE treatment period, and (3) post-VTE treatment period. The “pre-VTE treatment” period was defined as 100 days prior to the index date (regardless of the date of PAD diagnosis). The “during VTE treatment” period lasted from the index date (ie, the date of the VTE) until 4 months after (120 days), as current Dutch guidelines recommend treating VTEs for 3 months [20], whereafter an outpatient clinic visit is scheduled to discuss (dis)continuation or modification of ATT. One additional month was included in the treatment period definition to provide some flexibility in the treatment timeline. We performed sensitivity analyses to assess the robustness of our findings using a VTE treatment period definition of 180 and 200 days. The “after VTE treatment” period lasted from 4 months until 12 months after the index date. Within each treatment period, patients were classified in 10 different ATT groups according to the presence of at least one ATT prescription during that period (one prescription of that type of ATT was sufficient for classification in that category): low molecular weight heparin (LMWH), direct oral anticoagulant (DOAC) or a vitamin K antagonist (VKA) alone or in combination with antiplatelet therapy, antiplatelet therapy alone, VKA to DOAC switchers or vice versa concomitantly with or without antiplatelet therapy, or no prescription. LMWH prescriptions alongside DOAC or VKA were not classified as a separate category, as LMWHs are only prescribed immediately before a DOAC or as a bridging therapy with VKAs and are thus only prescribed for a short period of time [21].

### 2.4 Outcomes

The primary outcome was the use of ATT during the different treatment periods (before, during, and after VTE (treatment)), which is depicted by a longitudinal overview of (combinations of) ATT using an alluvial diagram. Secondly, one-year cumulative incidences of the following outcome events were established: arterial thromboembolism and its individual components (myocardial infarction, ischemic stroke, and transient ischemic attack, and other systemic arterial embolisms), clinically relevant bleeding events, progression of peripheral artery disease, MALE, and death. Arterial thromboembolic events and clinically relevant bleedings were identified by their corresponding ICD-10 codes (definitions are provided in Supplemental Material section 2). MALE included either (1) a performed revascularization, including all revascularization procedures registered during hospital admission, from alteplase to bypass surgery and thrombectomy, or (2) a minor and/or major lower limb amputation (Supplemental Material section 2). Revascularizations and amputations were identified based on the presence of their procedure codes in the data. Only the first events were included for each outcome.

### 2.5 Statistical analysis

The study population was characterized at index by proportion and frequency for binary indicators and median, lower and upper quartiles, as well as mean and standard deviation for continuous covariates. The cumulative incidences of arterial thromboembolic events, clinically relevant bleedings, progression of PAD, MALE and mortality were established for the complete cohort, as well as in subgroups based on clinically relevant risk factors: use of oral anticoagulation alone versus concomitant oral anticoagulation and antiplatelet therapy, Fontaine II PAD versus chronic limb-threatening ischemia (CLTI, ie, patients with Fontaine III or IV PAD), and in patients with and without diabetes mellitus. Cumulative incidences were all accounted for the competing risk of death using the Cumulative Incidence Competing Risk method. For an overall picture of survival, a Kaplan-Meier curve was established. All analyses are conducted in RStudio (R version 4.4.0). The manuscript was drafted according to the Reporting of Studies Conducted using Observational Routinely-collected health Data (RECORD) Statement (checklist Supplemental Material section 5). No funding was provided for the conduct of this research.

## 3 Results

### 3.1 Cohort characteristics

**Figure 1:**
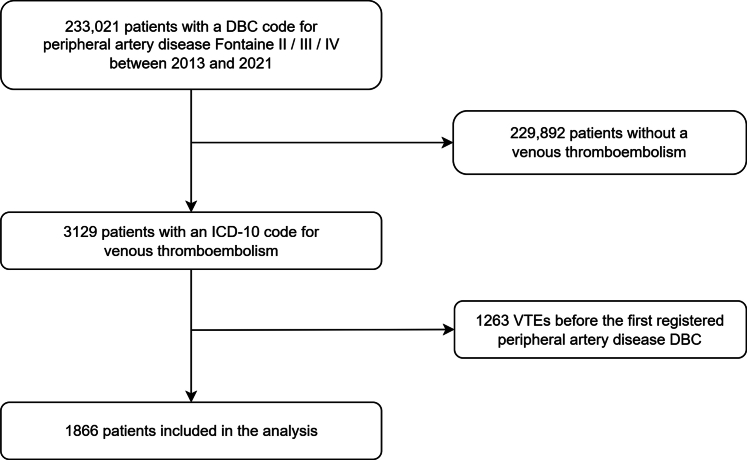
Flowchart of patient inclusion. DBC, Diagnose Behandel Combinatie (Diagnosis Treatment Combination); ICD, International Classification of Diseases.

**Figure 2:**
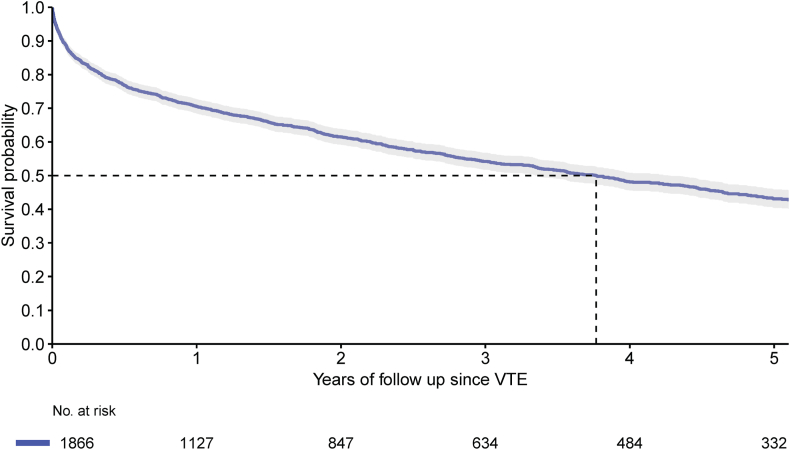
Kaplan-Meier curve of survival of the entire cohort.

Between January 1, 2013 and January 1, 2022, 233,021 patients had a diagnosis of PAD registered. Among those patients, 1866 had a hospital admission due to VTE registered after their first PAD diagnosis, and were included in the analysis (Figure 1); 42.2% were women, and the mean age was 71.7 years. At the time of the index VTE, most patients had Fontaine stage II PAD (66.1%), followed by Fontaine stage IV (20.6%) and stage III (13.3%). Most frequent comorbidities were hypertension (30.9%), diabetes mellitus (22.1%), and chronic obstructive pulmonary disease (16.8%) (Table). Median follow-up was 1.7 years (IQR: 0.4, 4.2). During the VTE treatment phase, 24% of patients died. Median survival was 3.8 years for the entire cohort (Figure 2), and 4.5 years and 2.8 years for Fontaine II and CLTI (Fontaine III and IV) patients (Supplemental Figure 1), respectively.

**Table:** Baseline characteristics.

Patient characteristics	Overall (*N* = 1866)
Sex, female, *n* (%)	787 (42.2)
Age, mean (SD)	71.7 (10.9)
Age group, y, *n* (%)	
18-50	65 (3.5)
50-60	180 (9.6)
60-70	470 (25.2)
70-80	670 (35.9)
80+	481 (25.8)
Immigration background, *n* (%)	
Native Dutch	1646 (88.2)
1st generation immigrants	106 (5.7)
2nd generation immigrants	114 (6.1)
Peripheral arterial disease stage, *n* (%)	
Stage II	1233 (66.1)
Stage III	248 (13.3)
Stage IV	385 (20.6)
Comorbidities, *n* (%)^a^	
Atrial fibrillation	120 (6.4)
Anemia	237 (12.7)
Asthma	65 (3.5)
Autoimmune disease	113 (6.1)
Coagulopathy	47 (2.5)
Chronic obstructive pulmonary disease	314 (16.8)
Chronic lung disease (other)	82 (4.4)
Stroke/transient ischemic attack	109 (5.8)
Diabetes mellitus	413 (22.1)
Congestive heart failure	161 (8.6)
Hypertension	577 (30.9)
Immune deficiency	13 (0.7)
Kidney disease	273 (14.6)
Liver disease	61 (3.3)
Major bleeding	139 (7.4)
Myocardial infarction	273 (14.6)
Other arterial thromboembolism	221 (11.8)
Valvular heart disease	102 (5.5)
Thyroid disease	42 (2.3)
History of venous thromboembolism	139 (7.4)

a Comorbidities are based on diagnoses that were registered during hospitalization in a Dutch hospital following the ICD-9 and ICD-10 classification during the 3 years prior to the index date. Other chronic lung diseases include bronchitis, lung emphysema, bronchiectasis, and acute infection of the upper airways. Other arterial thromboembolisms include arterial embolisms/thrombosis/occlusions located elsewhere than in cerebral, renal, coronary, mesenteric, pulmonary, or retinal arteries.

### 3.2 Treatment patterns

**Figure 3:**
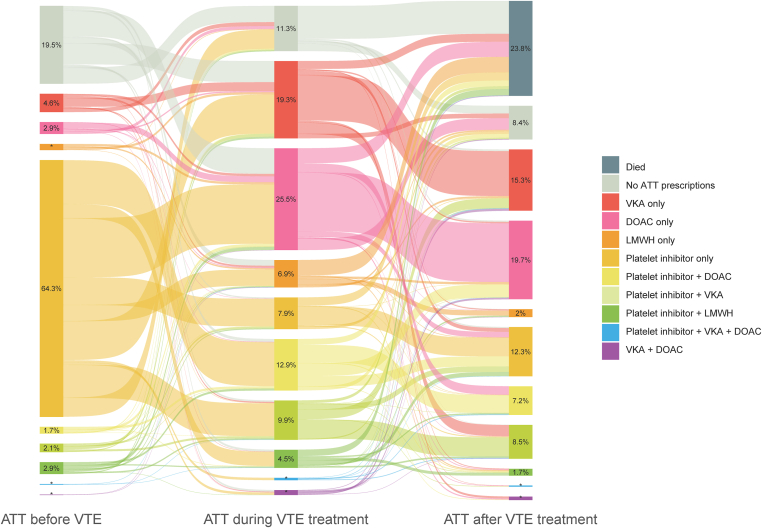
Alluvial diagram depicting ATT prescription patterns in patients with lower extremity peripheral artery disease. The ATT before VTE period lasted for 3 months before a venous thromboembolism, the “ATT during the VTE treatment period” until 120 days after the VTE occurred, and “ATT after VTE treatment” lasted from 120 days until 12 months after VTE. *N* = 1866 in all timeframes. ATT, antithrombotic therapy; DOAC, direct oral anticoagulant; LMWH, low molecular weight heparin; VKA, vitamin K antagonist; VTE, venous thromboembolism. ∗Numbers add up to 9 or less, and for data protection reasons, cannot be exported.

In the pre-VTE treatment period, 64% of the patients received antiplatelet therapy only, while 19% were not receiving any ATT (Figure 3). A VKA was dispensed to 14% of the patients, of whom 47% also received an antiplatelet agent during this period.

In the VTE treatment period, monotherapy DOACs were the most frequently dispensed type of ATT (25%), followed by vitamin K antagonists (19%), and LMWH alone (6.9%). Anticoagulation and a platelet inhibitor were dispensed to 27% of the patients, of whom 84% were already on antiplatelet therapy prior to the index VTE.

Treatment patterns from pre-treatment phase to the VTE treatment phase showed that most patients who did not receive any type of ATT prior to their VTE were treated for their VTE with therapeutic anticoagulation without addition of antiplatelet therapy (80%). After VTE, 25% of the prevalent VKA only users and 45% of the DOAC only users continued their ATT unmodified. Of the entire cohort, 11% did not receive any ATT in the 4 months following their VTE. Most of these untreated patients had used a platelet inhibitor (42%) or were not dispensed any type of ATT prior to their VTE (36%). Of the untreated patients, 73% died during the VTE treatment phase.

After the VTE treatment period, most patients who were dispensed only VKA or DOAC for their VTE continued on the same treatment during the 4 to 12 months after the VTE (58% in both groups). The proportion of patients having therapeutic anticoagulant dispensings together with a platelet inhibitor decreased from 27% to 18%, while 8.4% of the patients did not receive any ATT after VTE treatment. Only antiplatelet therapy was dispensed to 12% of the patients after the VTE treatment period.

During each treatment phase, a small proportion of patients switched from VKA to DOAC or vice versa, indicated by the presence of both VKA and DOAC dispensings during one treatment period. As the number of patients in these groups was less than 10, we were not able to report exact numbers due to data protection reasons.

### 3.3 Clinical events

**Figure 4:**
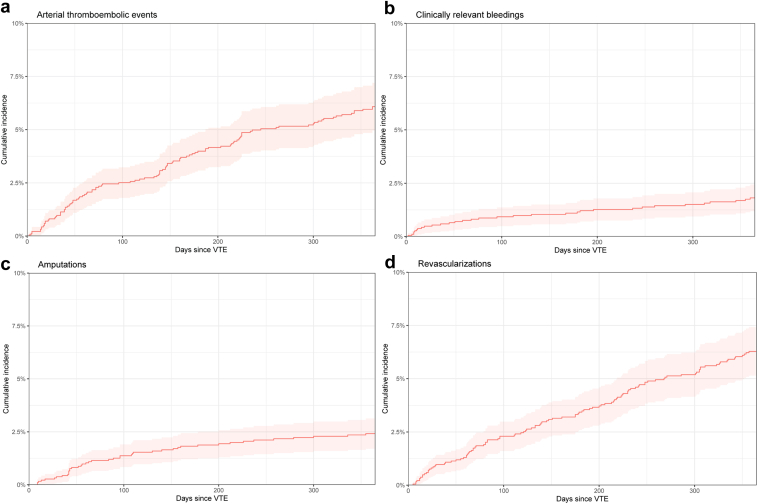
Cumulative incidences of first arterial thromboembolic events, clinically relevant bleedings, amputations, and revascularizations during the first year after venous thromboembolism occurred.

A total of 129 arterial thromboembolic events occurred in 107 patients. The cumulative incidence of a first arterial thromboembolic event within 1 year after VTE was 6.1% (95% CI, 4.9-7.3) (Figure 4). Most events were strokes or transient ischemic attacks (36%) or occurred in the lower limbs (33%), followed by myocardial infarctions (17%) and events in other anatomical sites (13%). Clinically relevant bleeding events occurred in 1.8% (95% CI, 1.2-2.4) of patients during the first year after the VTE.

The one-year cumulative incidences accounted for the competing risk of death of revascularizations and lower limb amputations were 6.3% (95% CI, 5.1-7.5) and 2.4% (95% CI, 1.6-3.2) in the entire cohort, respectively (Figure 4).

### 3.4 Subgroup analyses

Patients who were dispensed both anticoagulation and antiplatelet therapy during the VTE treatment period had higher one-year cumulative incidences of arterial thromboembolic events, clinically relevant bleedings, and MALE than patients who were dispensed anticoagulation only (Supplementary Table 1).

Patients with CLTI had higher one-year cumulative incidences of arterial thromboembolic events (7.3% [95% CI, 5.1-9.5] vs 5.4% [4.0-6.8]), clinically relevant bleedings (2.5% [95% CI, 1.3-3.7] vs 1.5% [95% CI, 0.7-2.3]), amputations (6.4% [95% CI, 4.4-8.4] vs N < 10) and revascularizations 11.6% (95% CI, 9.1-14.1) than patients with PAD Fontaine stage II (Supplementary Table 2).

Likewise, patients with diabetes mellitus had higher cumulative incidences of arterial thromboembolic events, clinically relevant bleedings, amputations, and revascularizations than patients without diabetes mellitus (Supplementary Table 3)

Of the patients with a history of VTE before their PAD diagnosis, 64% were dispensed an oral anticoagulant after the VTE treatment period, of whom 42% received antiplatelet therapy as well. After the VTE treatment period, 7% of the patients did not receive any ATT.

### 3.5 Sensitivity analyses

As a sensitivity analysis, we defined the VTE treatment period as 180 days instead of 120 days (Supplemental Figures 2 and 3). Consequently, the proportion of patients who died during the VTE treatment period changed from 24% to 26%. After VTE treatment, patients who only got antiplatelet therapy increased from 12% to 16%, and antiplatelet therapy dispensings in combination with either a VKA or DOAC decreased by 3.7% and 3.2%, respectively. Patients with no ATT dispensing after VTE treatment increased from 8.4% to 11.4%. Extending the treatment period from 180 to 200 days further increased the proportion of patients with no ATT and those dispensing a platelet inhibitor only (to 13% and 17%, respectively).

After excluding the patients with atrial fibrillation, similar patterns as in the entire population (with patients with atrial fibrillation included) were observed (Supplementary figure 4). The most substantial differences were in the period after the VTE treatment, where the proportion of patients who received no ATT increased from 8.4% to 11.6%, and the proportion that received only antiplatelet therapy increased from 12% to 16%.

No ATT dispensings were recorded for 71 patients (3.8%) during any of the treatment periods. Of these, 32 patients (45%) had an indication for a permanent residency in a nursing home at the time of the index date, of which the dispensing data are not in our database. Moreover, 20 patients (28%) died within two weeks after VTE occurred. For the other 11 patients, no reason for having no ATT dispensation was found.

## 4 Discussion

The goal of this study was to describe (1) current prescription patterns in patients with symptomatic PAD who develop VTE, (2) how ATT is further modified after the VTE treatment (lasting generally 3 to 6 months) has passed, and (3) incidences of arterial thromboembolic events, clinically relevant bleedings, and MALE after VTE occurred.

The main findings of this study are threefold: (1) VTE treatment patterns in patients with symptomatic PAD are considerably heterogenous, where a substantial proportion of patients was dispensed both anticoagulant and antiplatelet therapy during and after VTE treatment (28.3% and 17.5%, respectively), (2) patients with symptomatic PAD were undertreated, with almost 1 in 5 patients with symptomatic PAD not receiving any ATT prescription in the period before their VTE, and (3) the risks of adverse events, clinical events, and death were high.

The incidences of arterial thromboembolic events observed in our PAD cohort were consistent with those reported in other studies [[22], [23], [24]]. Patients using both anticoagulation and antiplatelet therapy during VTE treatment had a higher incidence of arterial thromboembolic events, revascularizations, and amputations compared with patients using anticoagulation only (Supplementary Table 1) These observations are likely a result of confounding by indication, such that patients with more severe disease were kept on antiplatelet therapy, and by virtue of the more severe disease, they had a higher incidence of adverse outcomes. While we cannot exclude the possibility of excess risk as a result of concomitant antiplatelet therapy, as recently observed in some clinical trials, there is no current plausible mechanism for that other than that patients may have stopped all ATT after a bleeding event [25,26].

Survival was lower in our cohort (median survival of 3.8 years) than in other PAD cohorts. In the VOYAGER-PAD trial [27], 3-year all-cause death rates were around 11%, while in the COMPASS trial, 5% to 6% of the patients died during a maximum follow-up of 3 years [22]. These differences can primarily be attributed to the overall health differences between “practice-based” and trial populations, and to our slightly higher proportion of CLTI patients, who have a higher mortality risk than Fontaine (I and) II patients [28]. Our mortality rate indeed aligns more with the mortality of 33% during 2.5 years of follow-up in the SWEDE-PAD trial, in which patients with PAD and an indication for endovascular treatment were included [29]. Moreover, our cohort consisted of patients with a VTE diagnosed in hospitals, missing a most likely limited number of patients with a deep vein thrombosis who were treated by the general practitioner only, resulting in a study population with more severe VTE than in the general PAD population, adding to the higher mortality rate found in our study. All in all, these high mortality rates underscore the severe burden of disease in the PAD population.

Our study population had high comorbidity rates, but these were still lower than the PAD population included in trials [22,27]. This can be attributed to our definition of comorbidities, for which we rely on all diagnoses registered during hospitalizations 3 years prior to the index date, acknowledging that not all comorbidities may have been registered during hospitalizations, in particular when treated by the general practitioner.

A small proportion of patients (*N* = 71; 3.8%) did not have any ATT dispensing during any of the treatment periods. In many of these patients (*N* = 32; 45%), this was probably due to their residency in a nursing home. We lack data on prescriptions during hospital admissions and of nursing home residents without an independent general practitioner, as our database only contains dispensing data from outpatient pharmacies. Other patients without ATT prescriptions were possibly in the last phase of life or hospitalized, as 20% of these patients died within 2 weeks after VTE, resulting in the absence of ATT dispensings. In total, in only 11 patients (0.6%) a clear reason could not be identified.

During the VTE treatment period, 27% of the patients were dispensed antiplatelet agents next to anticoagulants. The dosages of the prescribed anticoagulants were unavailable; however, it is likely that these were therapeutic during this period considering the standard of care for VTE treatment, and the VOYAGER-PAD trial, which led to the recommendation of concomitant use of low-dose rivaroxaban and antiplatelet therapy, was only published at the end of our study period and thus not yet implemented in Dutch hospitals during the study period [27]. Notably, the combination of therapeutic anticoagulation with antiplatelet agents is not routinely recommended for VTE management [20], increasing the bleeding risk in patients, and unnecessarily so in those without an indication for such combination [16,30]. Sensitivity analyses showed that after prolonging the VTE treatment period (from 3 to 6 months), the proportion of patients with concomitant dispensings of antiplatelet and anticoagulation after the VTE treatment period decreased, while the proportion of patients with antiplatelet alone and no ATT in all categories increased (Supplementary Figures 2 and 3). This is due to some patients having extended (6 months) treatment of their VTE, whereafter the therapeutic anticoagulation is discontinued. This is justified in provoked VTE; however, for unprovoked VTE, secondary prevention in these patients likely requires a more intensive approach than antiplatelet therapy alone can provide, given their increased risk of both venous and arterial thromboembolism (and MALE). Earlier studies have suggested the efficacy of low-dose rivaroxaban in combination with aspirin in the prevention of arterial thromboembolic events [22,27], however, they lack generalizability [31,32] and do not compare aspirin and add-on DOAC with P2Y12 inhibitors, while the P2Y12 inhibitors seem more potent than aspirin in preventing arterial events [33,34]. More recently, the EPIC-CAD trial showed an increased risk of bleeding without an effect on the reduction of thromboembolic complications with concomitant full-dose edoxaban and antiplatelet therapy compared with edoxaban alone in patients with stable coronary artery disease [35]. In an educational paper, May and Moll meticulously highlight the treatment challenges in patients with both arterial and venous thromboembolism and report possible treatment strategies [18]. In the acute phase, VTE should be treated with a therapeutic anticoagulant while discontinuing antiplatelet therapy (unless there is a strong indication for antiplatelet therapy such as a recent myocardial infarction or percutaneous transluminal angioplasty, which may be the case for some of the patients in our cohort on antiplatelet and anticoagulant therapy, but this was not part of our evaluation) [25,36]. For long-term secondary prevention of both venous and arterial events, patients with PAD who experience a VTE (not focusing on patients with an indication for therapeutic anticoagulation (such as recurrent [bypass] occlusions) may benefit from (lifelong) low-dose apixaban (2.5 mg) or rivaroxaban (10 mg), or possibly a combination of “baby-dose” rivaroxaban (2.5 mg) in combination with antiplatelet therapy, but more evidence is needed [37,38]. Treatment in these patients definitely needs more than a one-size-fits-all approach, and decisions on long-term treatment should be carefully weighted.

With the findings of our study, we cannot give a recommendation on the best long-term treatment of VTE in patients with PAD. However, based on the overview of current practice patterns, we can identify potential improvements in current care. First, we should take care that all patients with symptomatic PAD receive antiplatelet therapy to prevent arterial thromboembolic complications. This is currently not the case, reflected by the lack of any type of ATT dispensations in 19% of the patients during the 3 months prior to their VTE (which can only be explained in 9% of these patients (*N* = 33) by lack of prescription data from nursing home residents). Second, it is important to realize that except for temporary use after stenting (1-12 months) or some rare indications, simultaneous use of both antiplatelet therapy and therapeutic anticoagulation is almost never indicated, although current guidelines do recommend very low-dose rivaroxaban 2.5 mg and antiplatelet therapy combination for long-term secondary prevention [39,40]. This should be anticipated in patients already using antiplatelet therapy prior to their VTE by discontinuing the antiplatelet therapy upon starting therapeutic anticoagulation. Lastly, the limited survival and high incidences of arterial thromboembolic events, revascularizations, and amputations underscore the need for strict adherence to cardiovascular risk management, including lifestyle interventions and management of other cardiovascular risk factors, perhaps indicating intensified follow-up.

One of the strengths of our study is the use of electronic health care records data, reflecting the “real-life” treatment patterns in clinical practice. The nationwide coverage provided a sufficient sample size, allowing us to create a clear longitudinal overview of the current practice patterns in The Netherlands. Additionally, we included an unselected population with complete follow-up. However, the study also has some limitations. We may have missed patients with deep vein thrombosis who were primarily diagnosed and managed in primary care, particularly those with PAD Fontaine stage II, who in The Netherlands are often treated by general practitioners rather than by vascular surgeons. For the classification of comorbidities in the CBS data, we are dependent on the ICD-10 system. Diseases or interventions may be wrongly classified, though the extent of such misclassification is unknown. The registration for medical interventions changed in 2018 from manual to automatic registration, which may have influenced the estimation of cumulative incidences of MALE. However, this effect is likely minimal (Supplementary Figure 5 and 6). The use of ATC5 codes for ATT means we only have data on medication groups, without details on specific drugs or dosages. Moreover, the prescription data are based on dispenses at the pharmacy, and we have no information on the actual use of ATT by the patients. While prior nationwide Belgian and Dutch studies report 6.5% up to 40% nonadherence to anticoagulation in atrial fibrillation patients [[41], [42], [43], [44]], we expect lower rates of anticoagulation nonadherence in our study, as symptomatic VTE may encourage better adherence than preventive atrial fibrillation therapy. Progression of PAD could not be identified based on our pre-defined definition, because the total of DBCs with a Fontaine stage higher than the index DBC was lower than 10, and these data could not be exported from CBS due to privacy reasons. Lastly, the observational nature of our study design introduces confounding by indication, limiting our research to descriptive analyses only, while not being able to take into account the time-varying component of medical treatment.

To conclude, our study demonstrates heterogeneous treatment patterns of VTE in patients with PAD and a high risk of cardiovascular events and clinically relevant bleeding. Notably, a substantial proportion of patients received antiplatelet therapy and anticoagulation simultaneously, exposing them to an increased bleeding risk. Moreover, a large proportion of patients had no prescription of any type of ATT prior to their VTE, which suggests a need for greater clinical attention to prescribing antiplatelet therapy in patients with PAD and to monitor adherence. Our findings highlight the need for better adherence to clinical guidelines and improved integration of current knowledge into practice, which could facilitate consistent, high-quality care and ultimately improve patient outcomes.
