# Supplementary material for: Antithrombotic therapy in lower extremity peripheral artery disease patients with venous thromboembolism: a nationwide cohort study
Source: Res Pract Thromb Haemost. 2025 Dec 9;10(1):103291. doi: 10.1016/j.rpth.2025.103291 (PMC12818358; doi:10.1016/j.rpth.2025.103291)
Supplement: Supplementary Material [file mmc1.pdf]

Antithrombotic therapy use and clinical outcomes in peripheral artery disease patients with incident venous thromboembolism: a Dutch nationwide cohort study

Goedegebuur J, Butera E, Chen Q, Bikdeli B, Ageno W, Pola R, Porfidia A, Barco S,
Van der Vorst J.R., Cannegieter S.C., Klok F.A.

Supplemental material

Table of contents

Supplement 1: Detailed information of the datasets used in the study 3
Supplement 2: Code list for all variables 4
Supplement 3: Supplemental tables........ 8
Supplement 4: Supplemental figures 11
Supplement 5: RECORD statement........ 17

Supplement 1: Detailed information of the datasets used in the study

Variables	Datasets	Original variables used for identification	Comment
Personal identifier	All datasets	RINPERSOONS, RINPERSOON	Used to link different datasets
Birthdate	GBAPERSOONTAB	GBAGEBOORTEJAAR<br>GBAGEBOORTEMAAND<br>GBAGEBOORTEDAG	Directly identified by the variables
Sex		GBAGESLACHT	Directly identified by the variable
Immigration background		GBAGENERATIE	Directly identified by the variable
Medications	MEDICIJNTAB	ATC4	Directly identified by the variable;<br>The detailed codes used for identifying different medication are presented in Supplement 2.
Date of death	GBAOVERLIJDENTAB	GBADATUMOVERLIJDEN	Directly identified by the variable
Admission diagnoses	LBZBASISTAB	LBZICD10hoofddiagnose<br>LBZhoofdverrichting	Directly identified by the variable
Admission diagnoses	LBZDIAGNOSETAB	LBZicd10diag	Directly identified by the variable
PAD Diagnosis and stage	MSZPrestatiesvekttab	VEKTMSZDBCZorgproduct	Directly identified by the variable;<br>The detailed codes used for identifying different medication are presented in Supplement 2.
Revascularizations, amputations	MSZZorgactiviteitentab / LBZDiagnosetab	Zorgactiviteit (number) or LBZHoofdverrichting (CVV/CBV number)	Directly identified by the variable, using various codes for multiple activities (CBV until 2018, CVV from 2018 onwards and ZA-numbers)
Comorbidities	LBZBASISTAB	LBZICD10hoofddiagnose<br>LBZhoofdverrichting	Directly identified by the variable
Comorbidities	LMR	DEDIAG5	Directly identified by the variable

Supplement 2: Code list for all variables

Population definition

	Diseases	ICD-10
VTE	Deep vein thrombosis	I801,I802,I803
	Pulmonary embolism	I26
	Portal vein thrombosis	I81
	Budd-Chiari syndrome	I820
	Other VTE	H348, I808, I809, I821,I822,I823,I828,I829, K765, O223, O229, O871, O879, O882
	Cerebral venous sinus thrombosis	I636;I676; O225; O873

Diagnosis Treatment Combination codes for peripheral artery disease		
P.A.D II	Intermittent claudication	0303-04-38-0418
P.A.D III	Rest pain	0303-04-38-0419
P.A.D IV	Necrosis and/or gangrene of the limb	0303-04-38-0420

Exposure

ATC Codes antithrombotic agents		
Vitamin K antagonist	ATC	B01AA
Heparin group	ATC	B01AB
Platelet aggregation inhibitors	ATC	B01AC
Direct oral anticoagulant	ATC	B01AE (including B01AF)

Outcomes

Outcome variables	Comorbidities	ICD-10
ATE	Myocardial infarction	I21,I22
	Myocardial infarction (or history)	I21,I22,I23,I252
	Ischemic stroke	I63,I64, G46
	Ischemic stroke (or history)	I63,I64, G46, I693,I694
	Transient/central/other retinal artery occlusion	H340, H341, H342
	Transient ischemic attack	G45
	Systemic arterial thromboembolism	H340, H341, H342, I513, I74, K550
Bleeding	Major bleeding	D62,D683,H356,H431,I230,I312,I60,I61,I62,I850,I983,J942,K226,K250,K252,K254,K256,K260,K262,K264,K266,K270,K272,K274,K276,K280,K282,K284,K286,K290,K3180,K5520,K625,K6380,K661,K920,K921,K922,M250,N02,N837,N920,N921,N924,N938,N939,N950,R04,R31,R58,S064,S065,S066
	Intracranial hemorrhage	I60,I61,I62,S064,S065,S066
	Gastrointestinal bleeding	I850,I983,K226,K250,K252,K254,K256,K260,K262,K264,K266,K270,K272,K274,K276,K280,K282,K284,K286,K290,K625,K661, K920,K921,K922
	Intracranial hemorrhage without trauma	I60,I61,I62

Procedure codes

Revascularizations	Procedure names	Procedure health activity number (ZA number)
Acute revascularization (i.e. embolectomy or thrombolysis)	Embolectomy of abdominal blood vessels, Embolectomy of peripheral blood vessels, Mechanical thrombectomy, Thrombolysis using medication, Embolectomy of blood vessels in the head, neck, and base of the brain	33502, 33400, 33600, 80827, 80829
Percutaneous angioplasty (PTA)	PTA of cerebrovascular arteries, PTA of non-coronary peripheral arteries, PTA of occlusion of other non-coronary vessels, PTA of stenosis of other non-coronary vessels, PTA of non-coronary central arteries, PTA of the renal arter	33493, 33672, 80822, 80821, 33351, 33360
Reconstruction	Endovascular reconstruction of peripheral arteries using transplant, endarterectomy, endostent, or patch; Open reconstruction of a peripheral artery using transplant, endarterectomy, or patch with the use of an operating microscope; Open reconstruction of a peripheral artery using transplant, endarterectomy, or patch; Reconstruction of the aorta or its direct branches such as the renal arteries and iliac artery; Reconstruction of peripheral (arterial) vessels without a transplant; Insertion of an aorta bifurcation prosthesis and reconstruction of both renal arteries (open)	33668, 33669, 33670, 33555, 33554, 33450, 33341, 33685, 33684, 33561
Bypass	Axillo-bifemoral bypass, Axillo-femoral bypass, Cross-over of the subclavian artery or femoral artery, Femoro-popliteal bypass, Femoro-tibial bypass	33673, 33674, 33677, 33678, 33678, 33680
Endovascular procedure	Drug-eluting balloon; Endovascular stent, non-covered, for interventions on peripheral arteries and/or veins	190621, 190632

	Category minor/major	Site	ZA number	CBV
**Amputations**	Minor	Toe – shortening of metatarsal (MT)	NA	38746
		Toe – disarticulation or amputation	038791	38791
		Foot – removal of the 5th ray	NA	38747
		5th toe + MT5 except for the base	038795	38795
		Foot, Chopart/Lisfranc	038794	38794
		Foot – Chopart	NA	338792
		Toe – amputation/disarticulation of each additional toe	NA	338796
		Foot – tarsus	038790	38790
		Foot – exarticulation	038793	38793
	Major	Lower leg – amputation	038690	38690
		Amputation (Syme)	NA	338799B
		Amputation (Pirogoff) / Left / Right	NA	338799C / 338799L / 338799R
		Lower leg – exarticulation	038691	38691
		Upper leg – amputation	038590	38590
		Upper leg – exarticulation in the hip joint	NA	38951

	Category minor/major	Site	CVV
Amputations	Minor	**Amputation and disarticulation (including partial toe)**	**5845**
		Toe – amputation (including accessory toe)	58450
		Toe – exarticulation	58451
		Toe – ray excision	58452
		Toe – amputation/exarticulation NOS	58459
		**Foot – amputation and exarticulation**	**5846**
		Foot – mediotarsal amputation (Chopart)	58460
		Foot – tarsometatarsal amputation (Lisfranc)	58461
		Foot – transmetatarsal amputation	58462
		Foot – forefoot amputation	58463
		Foot – other amputation/exarticulation	58468
		Foot – amputation/exarticulation NOS	58469
	Major	**Amputation lower leg and exarticulation in ankle joint**	**5847**
		Lower leg – amputation	58470
		Amputation (Syme, ankle joint)	58471
		Ankle joint disarticulation	58472
		**Amputation thigh and exarticulation at the knee**	**5848**
		Thigh – amputation	58480
		Knee exarticulation	58482
		Hip exarticulation	58491

ICD-9/-10 codes used for comorbidities

Disease	ICD version	Code(s)
Asthma	10	J45; J46
	9	493
COPD	10	J44
	9	491; 492; 496
Other chronic lung disease	10	J41; J42; J43; J47; J6; J7
	9	494; 495; 50
Heart failure	10	I50
	9	428
Hypertension	10	I10; I11; I13; I15
	9	401; 402; 404; 405
AF	10	I48
	9	4273
Atherosclerosis	10	I20; I250; I251; I255; I258; I259; I70
	9	413; 4140; 4143; 4144; 4148; 4149; 4292; 440
Myocardial infarction (history)	10	I21; I22; I252
	9	410; 412; 4142
Rheumatic heart disease	10	I05; I06; I07
	9	3941; 395; 3971
Other valvular heart disease	10	I08; I34; I35; I36; I37; I38; I39; Z952
	9	3940; 3942; 3949; 396; 3970; 424; V433
peripheral artery disease	10	I739
	9	4439

Liver disease	10	B15; B16; B17; B18; B19; D684; I982; I983; K70; K71; K72; K73; K74; K75; K76; K77; Z944
	9	070; 4560; 4561; 4562; 571; 5722; 5723; 5724; 5728; 5731; 5732; 5733; 5735; V427
Diabetes	10	E10; E11; E12; E13; E14
	9	250
Thyroid disease	10	E00; E01; E02; E03; E04; E05; E06; E07
	9	240; 241; 242; 243; 244; 245; 246
Kidney disease	10	I12; N01; N02; N03; N04; N05; N06; N07; N08; N11; N12; N14; N150; N158; N159; N16; N18; N19; N25; N26; Q60; Q611; Q612; Q613; Q614; Q615; Q618; Q619; Z49; Z940
	9	403; 581; 582; 583; 585; 586; 587; 588; 5900; 7530; 7531; V420; V451; V56
Anemia	10	D5; D60; D61; D63; D64
	9	280; 281; 282; 283; 2840; 2848; 2849; 2850; 2852; 2858; 2859
Coagulopathy	10	D65; D66; D67; D680; D681; D682; D683; D685; D686; D688; D689; D69; D684
	9	286; 287
CVA/TIA (history)	10	G45; H340; H341; H342; I63
	9	3623; 434; 435; 436
(Other) arterial thromboembolism	10	I74
	9	444
VTE (history)	10	I26; I820; I822; I823; I828; I829
	9	4151; 452; 4530; 4532; 4533; 4534; 4536; 4538; 4539
Major bleeding	10	D62; D683; H313; H356; H431; H450; I312; I60; I61; I62; I850; I983; J942; K228; K250; K252; K254; K256; K260;K262;K264;K266; K270; K272; K274; K276; K280; K282; K284; K286; K290; K625; K661; K920; K921; K922; N02; N938; N939; N950; M250; R04; R31; R58; S064; S065; S066
	9	2851, 2878, 2879, 4230, 430, 431, 432, 4560, 4590, 5310, 5312, 5314, 5316, 5320, 5322, 5324, 5326, 5330, 5332, 5334, 5336, 5340, 5342, 5344, 5346, 5693, 578, 5967, 5997, 6271, 7191, 7847, 7848, 7863, 8524
Parkinsons disease	10	F023; G20
	9	3320
Alzheimers disease	10	F00; G30
	9	3310
Immune deficiency	10	D80; D81; D82; D83; D84; D89
	9	2790; 2791; 2792; 2793; 2798; 2799
Autoimmune autoinflammatory disease	10	D86; E271; G35; K50; K51; K900; M05; M06; M07; M08; M09; M30; M31; M32; M33; M34; M35
	9	135; 2554; 2794; 340; 556; 5790; 6960; 710; 714; 725
Malignant tumor	10	C
	9	14; 15; 16; 18; 19; 20; 17

Supplement 3: Supplemental tables

Supplemental Table 1. Cumulative incidence of events accounted for competing risk of death at time = 90 days, 180 days and 365 days, *stratified for VTE Treatment: Anticoagulation alone (AC) versus Anticoagulation+antiplatelet therapy (AC+APT).* CI = confidence interval.

	Arterial thromboembolic events		Clinically relevant bleedings	
	AC only (N = 952)	AC + APT (N = 508)	AC only (N = 952)	AC + APT (N = 508)
**Time (days)**	Cumulative incidence (95% CI)	Cumulative incidence (95% CI)	Cumulative incidence (95% CI)	Cumulative incidence (95% CI)
**90**	1.5% (0.7 - 2.3)	3.8% (2.0 - 5.6)	N < 10	N < 10
**180**	3.0% (1.8 - 4.2)	5.0% (3.0 - 7.0)	N < 10	N < 10
**365**	4.5% (3.1 - 5.9)	7.2% (4.8 - 9.6)	1.6% (0.8 - 2.4)	2.7% (1.3 - 4.1)

	Amputations		Revascularizations	
	AC only (N = 952)	AC + APT (N = 508)	AC only (N = 952)	AC + APT (N = 508)
**Time (days)**	Cumulative incidence (95% CI)	Cumulative incidence (95% CI)	Cumulative incidence (95% CI)	Cumulative incidence (95% CI)
**90**	N < 10	N < 10	1.6% (0.8 - 2.4)	3.0% (1.4 - 4.6)
**180**	1.3% (0.5 - 2.1)	2.4% (1.0 - 3.8)	2.9% (1.7 - 4.1)	5.0% (3.0 - 7.0)
**365**	1.5% (0.7 - 2.3)	3.3% (1.7 - 4.9)	5.1% (3.7 - 6.5)	10.0% (7.3 - 12.7)

Supplemental Table 2. Cumulative incidence of events accounted for competing risk of death at time = 90 days, 180 days and 365 days, *stratified for Fontaine II versus CLTI (=FontaineIII+IV) peripheral artery disease.* CI = confidence interval.

	Arterial thromboembolic events		Clinically relevant bleedings	
	Fontaine II (N = 1233)	CLTI (Fontaine III+IV) (N = 633)	Fontaine II (N = 1233)	CLTI (Fontaine III+IV) (N = 633)
**Time (days)**	Cumulative incidence (95% CI)	Cumulative incidence (95% CI)	Cumulative incidence (95% CI)	Cumulative incidence (95% CI)
**90**	2.5% (1.7 - 3.3)	2.4% (1.2 - 3.6)	N <10	N < 10
**180**	3.7% (2.7 - 4.7)	4.6% (3.0 - 6.2)	0.9% (0.5 - 1.3)	N < 10
**365**	5.4% (4.0 - 6.8)	7.3% (5.1 - 9.5)	1.5% (0.7 - 2.3)	2.5% (1.3 - 3.7)

	Amputations		Revascularizations	
	Fontaine II (N = 1233)	CLTI (Fontaine III+IV) (N = 633)	Fontaine II (N = 1233)	CLTI (Fontaine III+IV) (N = 633)
**Time (days)**	Cumulative incidence (95% CI)	Cumulative incidence (95% CI)	Cumulative incidence (95% CI)	Cumulative incidence (95% CI)
**90**	N < 10	3.4% (2.0 - 4.8)	0.8% (0.2 - 1.4)	4.6% (3.0 - 6.2)
**180**	N < 10	5.0% (3.2 - 6.8)	1.7% (0.9 - 2.5)	6.8% (4.8 - 8.8)
**365**	N < 10	6.4% (4.4 - 8.4)	3.6% (2.4 - 4.8)	11.6% (9.1 - 14.1)

Supplemental Table 3. Cumulative incidence of events accounted for competing risk of death at time = 90 days, 180 days and 365 days, *stratified for Diabetes Mellitus history yes or no*. CI = confidence interval.

	**Arterial thromboembolic events**		**Clinically relevant bleedings**	
	Diabetes Mellitus (N = 413)	No Diabetes Mellitus (N = 1453)	Diabetes Mellitus (N = 413)	No Diabetes Mellitus (N = 1453)
**Time (days)**	Cumulative incidence (95% CI)	Cumulative incidence (95% CI)	Cumulative incidence (95% CI)	Cumulative incidence (95% CI)
**90**	3.5% (1.7 - 5.3)	2.2% (1.4 - 3.0)	N < 10	N < 10
**180**	5.5% (3.1 - 7.9)	3.6% (2.6 - 4.6)	2.5% (0.9 4.1)	0.8% (0.4 - 1.2)
**365**	9.1% (6.2 - 12.0)	5.2% (4.0 - 6.4)	3.9% (1.9 - 5.9)	1.2% (0.6 - 1.8)

	**Amputations**		**Revascularizations**	
	Diabetes Mellitus (N = 413)	No Diabetes Mellitus (N = 1453)	Diabetes Mellitus (N = 413)	No Diabetes Mellitus (N = 1453)
**Time (days)**	Cumulative incidence (95% CI)	Cumulative incidence (95% CI)	Cumulative incidence (95% CI)	Cumulative incidence (95% CI)
**90**	N < 10	1.0% (0.4 - 1.6)	2.5% (0.9 - 4.1)	2.0% (1.2 - 2.8)
**180**	3.5% (1.7 - 5.3)	1.4% (0.8 - 2.0)	3.5% (1.7 - 5.3)	3.4% (2.4 - 4.4)
**365**	5.2% (3.0 - 7.4)	1.6% (1.0 - 2.2)	7.9% (5.2 - 10.6)	5.9% (4.7 - 7.1)

Supplement 4: Supplemental figures

Supplemental Figure 1. Kaplan Meier curve stratified for Fontaine stage (Fontaine II (blue) versus chronic limb-threatening ischemia (pink))

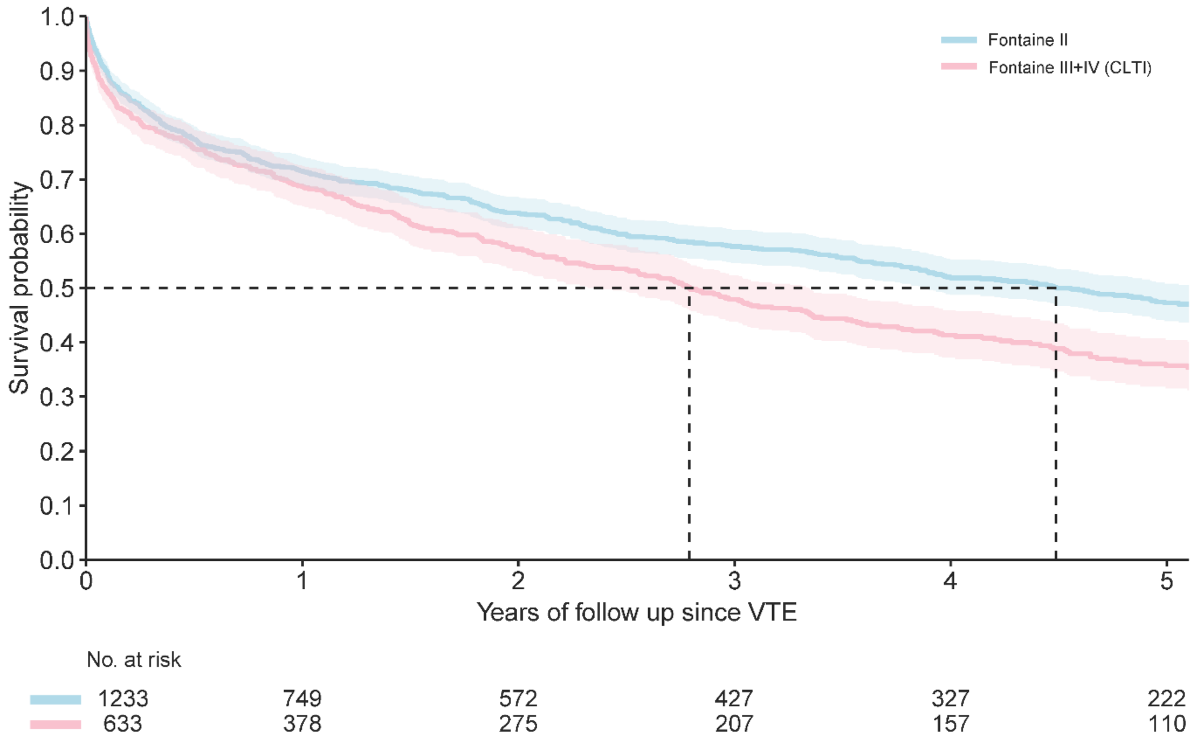

**Median survival**	
Fontaine II	4.5 years
Fontaine III+IV (CLTI)	2.8 years

Supplemental Figure 2.

Alluvial plot depicting ATT prescription patterns in patients with peripheral artery disease (PAD) 3 months before a venous thromboembolism, during the VTE treatment period (until 180 days after the VTE occurred) and after the VTE treatment (in the period 6 months until 12 months after VTE). N = 1866 in all timeframes.
* numbers add up to 9 or less and for data protection reasons, cannot be exported.

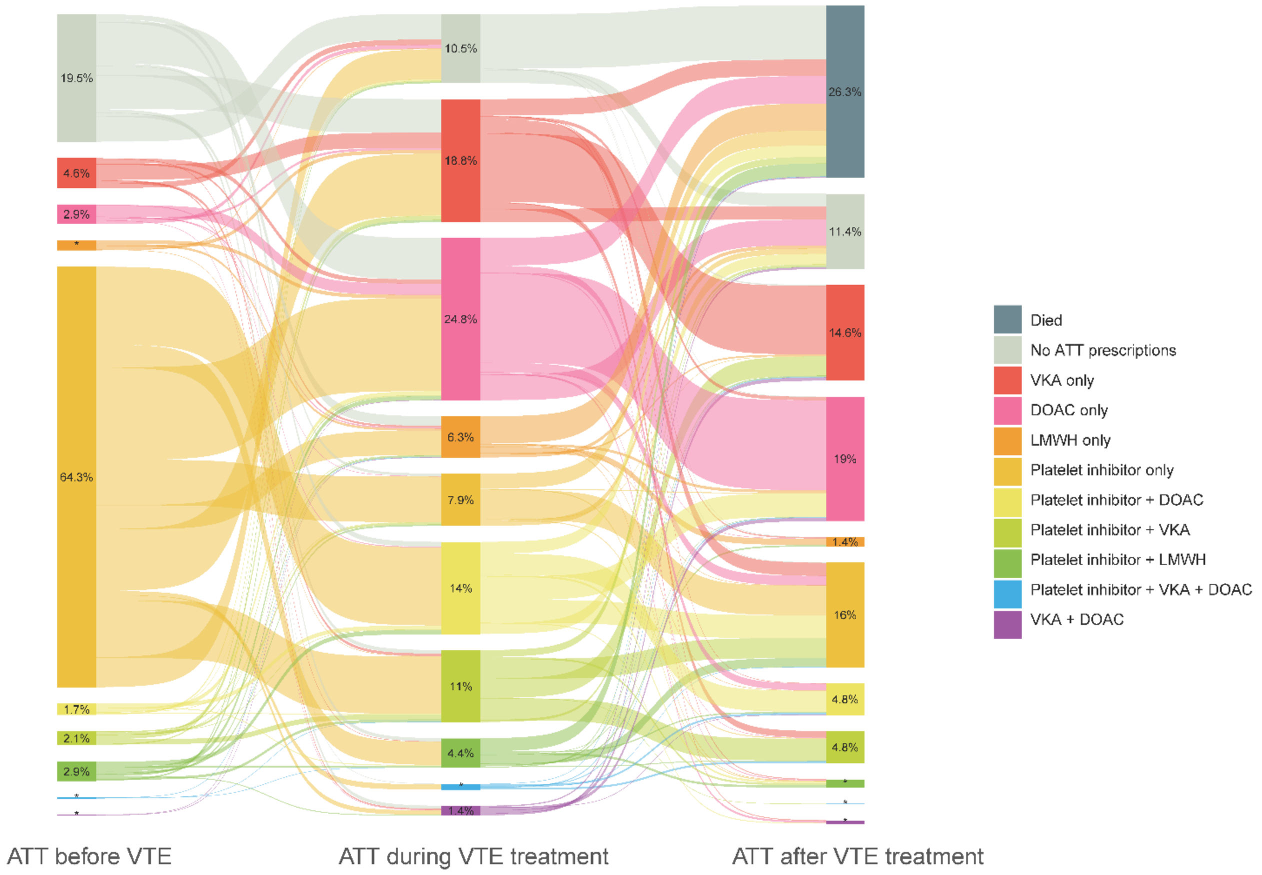

Supplemental Figure 3.

Alluvial plot depicting ATT prescription patterns in patients with peripheral artery disease (PAD) 3 months before a venous thromboembolism, during the VTE treatment period (until 200 days after the VTE occurred) and after the VTE treatment (in the period 200 days until 12 months after VTE). N = 1866 in all timeframes.
* numbers add up to 9 or less and for data protection reasons, cannot be exported.

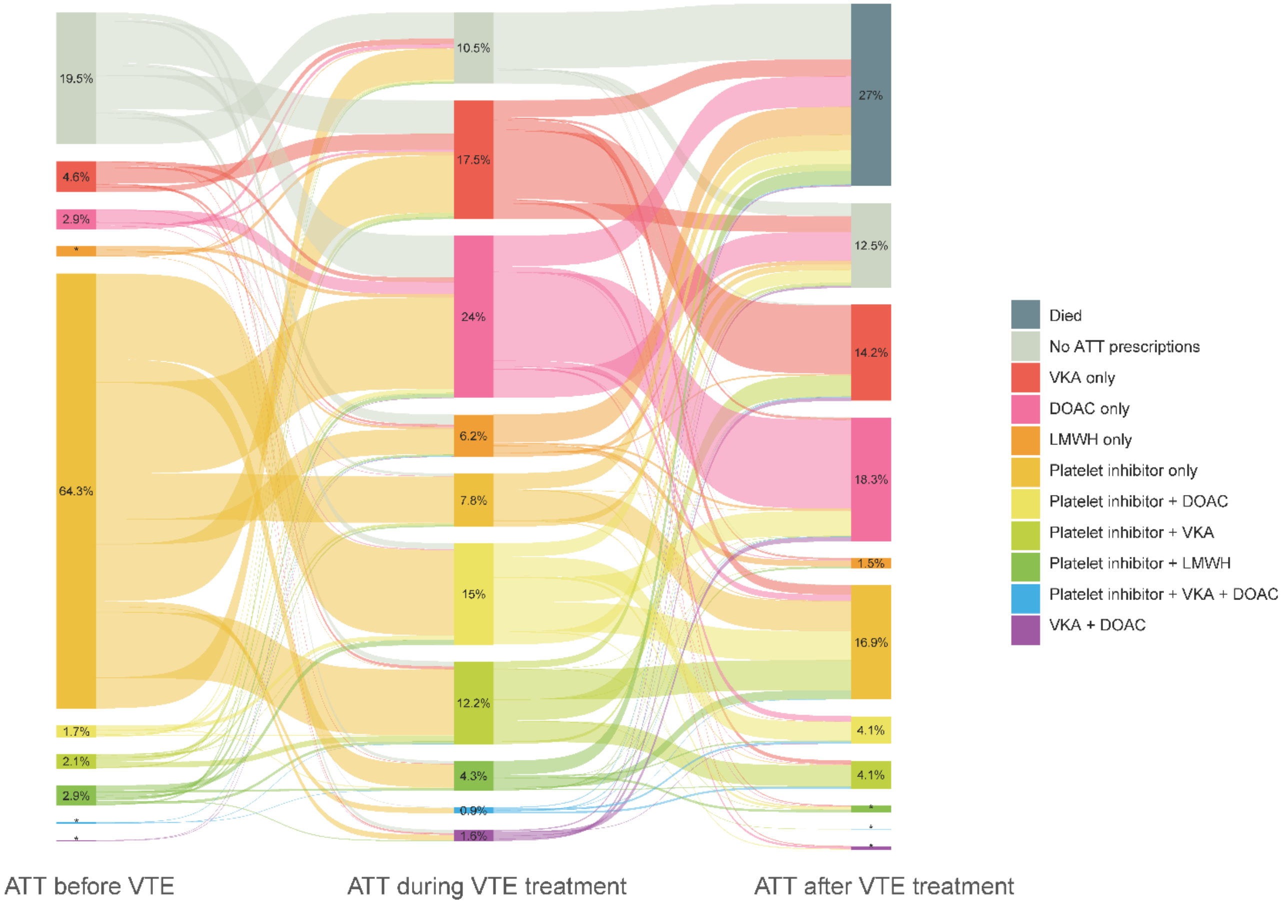

Supplemental Figure 4. Alluvial plot depicting ATT prescription patterns in patients with peripheral artery disease (PAD) 3 months before a venous thromboembolism, during the VTE treatment period (until 120 days after the VTE occurred) and after the VTE treatment (in the period 120 days until 12 months after VTE), excluding all patients with a history of atrial fibrillation. N = 1746 in all timeframes.
* numbers add up to 9 or less and for data protection reasons, cannot be exported.

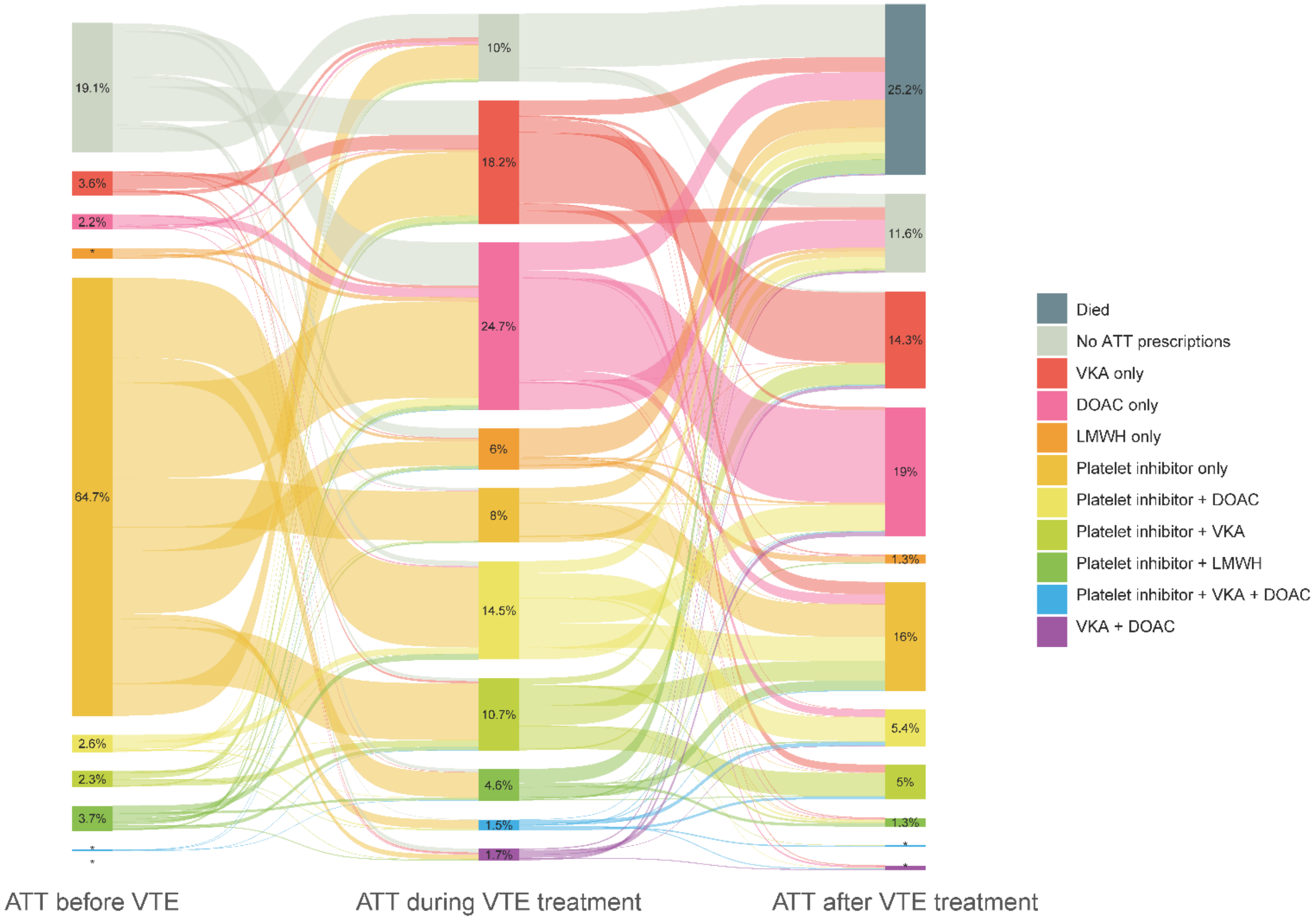

Supplemental Figure 5. Cumulative incidence curve of amputations of all patients with a VTE on or after January 1st 2018, accounted for the competing risk of death.

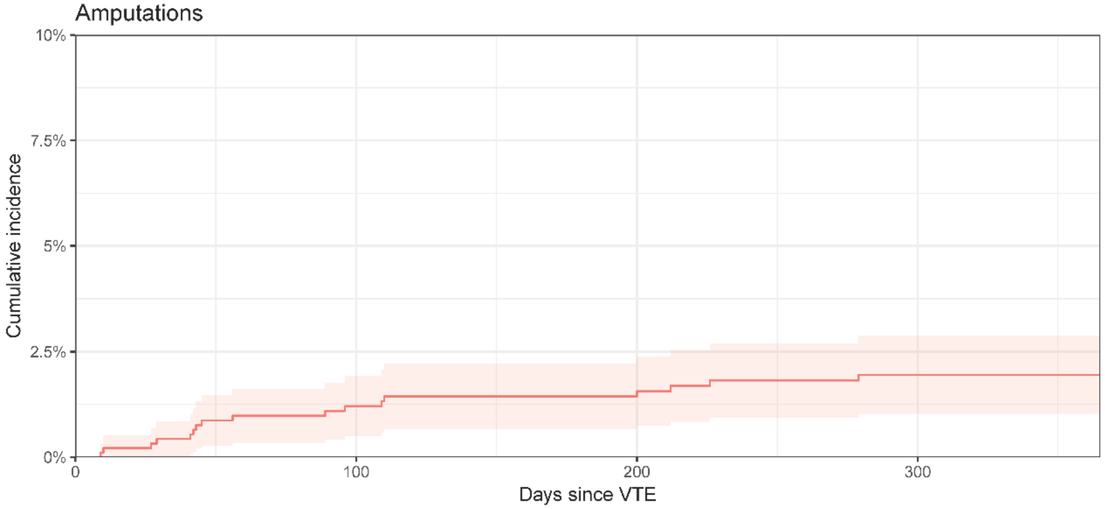

Supplemental Figure 6. Cumulative incidence curve of revascularizations of all patients with a VTE on or after January 1st 2018, accounted for the competing risk of death.

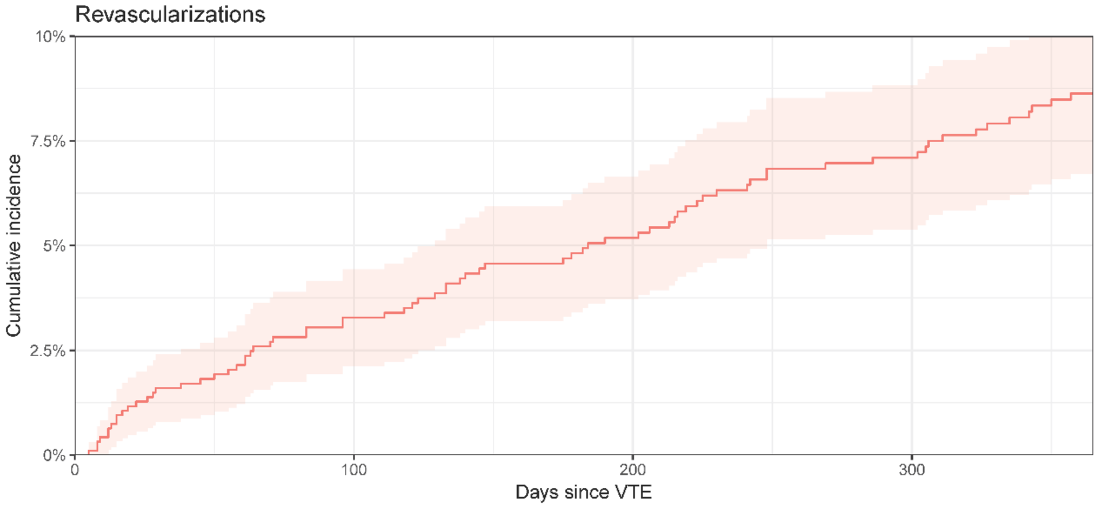

Supplement 5: RECORD statement

The RECORD statement – checklist of items, extended from the STROBE statement, that should be reported in observational studies using routinely collected health data.

	Item No.	STROBE items	Location in manuscript where items are reported	RECORD items	Location in manuscript where items are reported
**Title and abstract**					
	1	(a) Indicate the study's design with a commonly used term in the title or the abstract (b) Provide in the abstract an informative and balanced summary of what was done and what was found	Title and abstract	RECORD 1.1: The type of data used should be specified in the title or abstract. When possible, the name of the databases used should be included.<br><br>RECORD 1.2: If applicable, the geographic region and timeframe within which the study took place should be reported in the title or abstract.<br><br>RECORD 1.3: If linkage between databases was conducted for the study, this should be clearly stated in the title or abstract.	Abstract – methods<br><br>Abstract – methods<br><br>N.A.
**Introduction**					
Background rationale	2	Explain the scientific background and rationale for the investigation being reported	Introduction		
Objectives	3	State specific objectives, including any prespecified hypotheses	End of the introduction		
**Methods**					

Study Design	4	Present key elements of study design early in the paper	Methods – 1st paragraph		
Setting	5	Describe the setting, locations, and relevant dates, including periods of recruitment, exposure, follow-up, and data collection	Methods		
Participants	6	*(a) Cohort study* - Give the eligibility criteria, and the sources and methods of selection of participants. Describe methods of follow-up<br>*Case-control study* - Give the eligibility criteria, and the sources and methods of case ascertainment and control selection. Give the rationale for the choice of cases and controls<br>*Cross-sectional study* - Give the eligibility criteria, and the sources and methods of selection of participants<br><br>*(b) Cohort study* - For matched studies, give matching criteria and number of exposed and unexposed<br>*Case-control study* - For matched studies, give matching criteria and the number of controls per case	(a) Methods – 2nd paragraph<br><br>(b) N.A.	RECORD 6.1: The methods of study population selection (such as codes or algorithms used to identify subjects) should be listed in detail. If this is not possible, an explanation should be provided.<br><br>RECORD 6.2: Any validation studies of the codes or algorithms used to select the population should be referenced. If validation was conducted for this study and not published elsewhere, detailed methods and results should be provided.<br><br>RECORD 6.3: If the study involved linkage of databases, consider use of a flow diagram or other graphical display to demonstrate the data linkage process, including the number of individuals with linked data at each stage.	Supplements<br><br>N.A.<br><br>N.A.
Variables	7	Clearly define all outcomes, exposures, predictors, potential confounders, and effect modifiers.	Methods 3rd and 4th paragraph	RECORD 7.1: A complete list of codes and algorithms used to classify exposures, outcomes, confounders, and effect modifiers should be provided. If	Supplement

		Give diagnostic criteria, if applicable.		these cannot be reported, an explanation should be provided.	
Data sources/ measurement	8	For each variable of interest, give sources of data and details of methods of assessment (measurement).<br>Describe comparability of assessment methods if there is more than one group	Supplement		
Bias	9	Describe any efforts to address potential sources of bias	Discussion – last alinea		
Study size	10	Explain how the study size was arrived at	Figure 1		
Quantitative variables	11	Explain how quantitative variables were handled in the analyses. If applicable, describe which groupings were chosen, and why	N.A.		
Statistical methods	12	(a) Describe all statistical methods, including those used to control for confounding<br>(b) Describe any methods used to examine subgroups and interactions<br>(c) Explain how missing data were addressed<br>(d) *Cohort study* - If applicable, explain how loss to follow-up was addressed<br>*Case-control study* - If applicable, explain how matching of cases and controls was addressed<br>*Cross-sectional study* - If applicable, describe analytical	(a) + (b) + (e) Methods – last paragraph<br>(c) + (d) Methods – second paragraph		

		methods taking account of sampling strategy<br>(e) Describe any sensitivity analyses			
Data access and cleaning methods		..		RECORD 12.1: Authors should describe the extent to which the investigators had access to the database population used to create the study population.<br><br>RECORD 12.2: Authors should provide information on the data cleaning methods used in the study.	Methods – first Alinea<br><br>N.A.
Linkage		..		RECORD 12.3: State whether the study included person-level, institutional-level, or other data linkage across two or more databases. The methods of linkage and methods of linkage quality evaluation should be provided.	N.A.
**Results**					
Participants	13	(a) Report the numbers of individuals at each stage of the study (*e.g.*, numbers potentially eligible, examined for eligibility, confirmed eligible, included in the study, completing follow-up, and analysed)<br>(b) Give reasons for non-participation at each stage.<br>(c) Consider use of a flow diagram	Figure 1.	RECORD 13.1: Describe in detail the selection of the persons included in the study (*i.e.,* study population selection) including filtering based on data quality, data availability and linkage. The selection of included persons can be described in the text and/or by means of the study flow diagram.	Figure 1 and second paragraph of the methods section
Descriptive data	14	(a) Give characteristics of study participants (*e.g.*, demographic, clinical, social) and information on			

		exposures and potential confounders<br>(b) Indicate the number of participants with missing data for each variable of interest<br>(c) *Cohort study* - summarise follow-up time (*e.g.*, average and total amount)			
Outcome data	15	*Cohort study* - Report numbers of outcome events or summary measures over time<br>*Case-control study* - Report numbers in each exposure category, or summary measures of exposure<br>*Cross-sectional study* - Report numbers of outcome events or summary measures	Results – first paragraph		
Main results	16	(a) Give unadjusted estimates and, if applicable, confounder-adjusted estimates and their precision (e.g., 95% confidence interval). Make clear which confounders were adjusted for and why they were included<br>(b) Report category boundaries when continuous variables were categorized<br>(c) If relevant, consider translating estimates of relative risk into absolute risk for a meaningful time period	Results		

Other analyses	17	Report other analyses done—e.g., analyses of subgroups and interactions, and sensitivity analyses	Results – last paragraph		
**Discussion**					
Key results	18	Summarise key results with reference to study objectives	Discussion – first paragraph		
Limitations	19	Discuss limitations of the study, taking into account sources of potential bias or imprecision. Discuss both direction and magnitude of any potential bias	Discussion – paragraph before the conclusion	RECORD 19.1: Discuss the implications of using data that were not created or collected to answer the specific research question(s). Include discussion of misclassification bias, unmeasured confounding, missing data, and changing eligibility over time, as they pertain to the study being reported.	Discussion – paragraph before the conclusion
Interpretation	20	Give a cautious overall interpretation of results considering objectives, limitations, multiplicity of analyses, results from similar studies, and other relevant evidence	Conclusion (last paragraph of discussion)		
Generalisability	21	Discuss the generalisability (external validity) of the study results	Discussion		
**Other Information**					
Funding	22	Give the source of funding and the role of the funders for the present study and, if applicable, for the original study on which the present article is based	Methods – last sentence		
Accessibility of protocol, raw data, and		..		RECORD 22.1: Authors should provide information on how to access any supplemental information such as the	Protocol and raw code may be requested from

programming code				study protocol, raw data, or programming code.	the corresponding author – data is not available due to privacy reasons from the Statistics Netherlands.

*Reference: Benchimol EI, Smeeth L, Guttmann A, Harron K, Moher D, Petersen I, Sørensen HT, von Elm E, Langan SM, the RECORD Working Committee. The REporting of studies Conducted using Observational Routinely-collected health Data (RECORD) Statement. *PLoS Medicine* 2015; in press.
